# Traditional healers use of personal protective equipment: a qualitative study in rural South Africa

**DOI:** 10.1186/s12913-020-05515-9

**Published:** 2020-07-15

**Authors:** Carolyn M. Audet, Elisa Gobbo, Daniel E. Sack, Elise M. Clemens, Sizzy Ngobeni, Mevian Mkansi, Muktar H. Aliyu, Ryan G. Wagner

**Affiliations:** 1 Department of Health Policy, Vanderbilt University Medical Center, 2525 West End Avenue, Suite 1200, Nashville, TN 37203 USA; 2 School of Medicine, Vanderbilt University, 1161 21st Ave S # D3300, Nashville, TN 37232 USA; 3 Vanderbilt University, 2201 West End Ave, Nashville, TN 37235 USA; 4 MRC/Wits Rural Public Health and Health Transitions Research Unit, School of Public Health, Faculty of Health Sciences, University of the Witwatersrand, 7 York Rd, Parktown, Johannesburg, 2193 South Africa

**Keywords:** Traditional healers, South Africa, HIV/AIDS, Personal protective equipment, Blood exposure

## Abstract

**Background:**

Traditional healers are frequently exposed to hepatitis B virus (HBV), hepatitis C virus (HCV), and human immunodeficiency virus (HIV) through the widespread practice of traditional “injections”, in which the healer performs dozens of subcutaneous incisions using a razor blade to rub herbs directly into bloodied tissue. An average healer in Agincourt, a rural northeastern sub-district in Mpumalanga province, South Africa, experiences approximately 1500 occupational blood exposures over the course of their lifetime. Healers in Agincourt have an HIV prevalence of 30% compared to 19% in the general population, and healers who report exposure to patient blood have an adjusted 2.4-fold higher odds of being HIV-positive than those with no exposure. Although research on appropriate PPE use has been well documented for allopathic care providers, little is known about the practices of traditional healers.

**Methods:**

This qualitative study was conducted with 30 traditional healers who practice in the rural Bushbuckridge sub-district of Mpumalanga province, northeastern South Africa. We elicited traditional healer attitudes towards glove use during traditional treatments – including patient baths, injections, or other treatments that exposed healers to patient blood or open sores.

**Results:**

While 90% of healers reported using latex gloves during some treatments, the majority do not use them regularly. Most employ a combination of gloves, plastic shopping bags, bread bags, paper, and sticks to prevent blood exposure. Healers reported plastic bags slipping or breaking during procedures, exposing them to patient blood. Only three healers consistently used gloves, regardless of the cost.

**Conclusions:**

Inadequate PPE use and high HIV prevalence make traditional healers particularly susceptible to contracting HIV in rural South Africa. Despite positive attitudes, consistent glove use remains low due to financial constraints and glove availability. Addressing issues of accessibility and cost of gloves for traditional healers could have a significant impact on the adherence to PPE and, in turn, reduce new HIV infections among this high-risk group.

## Background

In 2018, 7700,00 people were living with HIV in South Africa, and 240,000 were newly infected with the virus [1]. UNAIDS has identified several groups of high-risk individuals, including men who have sex with men, sex workers, and adolescent girls, which have garnered substantial efforts to eliminate transmission and improve uptake of testing and treatment services [2]. Given the pervasiveness of HIV in the South African population, individuals regularly exposed to potentially HIV-positive blood, including health care providers, are likely at increased risk for exposure to HIV. Studies of occupational exposure in two South African hospitals revealed that 55 and 64% of medical interns reported at least one exposure to patient blood despite access to personal protective equipment (PPE), including latex gloves and sharps disposal containers [3, 4]. While in general parenteral exposure carries a low-risk for the acquisition of HIV, with a per-act probability of 23 per 10,000 exposures for a percutaneous needle stick, [5] repeated exposure to HIV-positive blood will increase the risk of acquiring the virus. This is especially true in contexts where PPE is not available or used less regularly, as may be the case for many of South Africa’s traditional healers.

Traditional Healers are an understudied group of health care providers that are largely unregulated, independent providers who perform health care services in the community. Within sub-Saharan Africa (SSA), traditional healers are frequently exposed to hepatitis B virus (HBV), hepatitis C virus (HCV), and human immunodeficiency virus (HIV) through the widespread practice of traditional “injections”, in which the healer performs dozens of subcutaneous incisions using a razor blade to rub herbs directly into bloodied tissue [6–9]. There are an estimated 2 million traditional healers in SSA, [10] with more than 200,000 practicing in South Africa [11]. Between 60 and 98% of healers regularly perform these highly sought after procedures [12, 13] on people with chronic disease, including those living with HIV [9, 14, 15]. An average healer in Agincourt, a rural northeastern sub-district in Mpumalanga province, South Africa, experiences approximately 1500 occupational blood exposures over the course of their lifetime [9]. Healers in Agincourt have an HIV prevalence of 30% compared to 19% in the general population [16]. Healers who report exposure to patient blood have an adjusted 2.4-fold higher odds of being HIV-positive than those with no exposure [17]. and, as such, may represent an unrecognized high-risk population who may be transmitting the virus to sexual partners, their children, other healers (94% of healers receive “injections”), and/or to their patients (via cuts/abrasions on their hands during “injections”). While HIV prevalence data is not available, to our knowledge, from healer’s practices in other parts of the country, the practice of “injections” is common across SSA. The risk of HIV acquisition by healers is driven by the underlying prevalence of HIV among the population of their patients and their correct use of PPE during treatments.

The use of PPE is an evidence-based practice that prevents infection by creating an impermeable barrier between the patient’s body fluids and the health provider, but PPE is only effective if used regularly and correctly. Although research on appropriate PPE use has been well documented for allopathic care providers, [18–24] little is known about the practices of traditional healers. This study aims to gain an understanding of traditional healers’ use of PPE, specifically the use of gloves or other forms of protection that prevention patient blood from getting in contact with bare skin.

## Methods

### Study location

This study was conducted with traditional healers who practice in the rural Bushbuckridge sub-district of Mpumalanga province, northeastern South Africa. The Agincourt Health and Socio-demographic Surveillance System (HDSS), an important site for demographic and population health-related research since 1992, [16, 25–28] includes 120,000 inhabitants and comprises 31 villages and approximately 21,000 households – served by one primary health center, eight local clinics, and three district level hospitals just outside the site’s borders. Most of the inhabitants are xiTsonga and roughly one-third of the HDSS population are Mozambican refugees who immigrated to South Africa during the 1980 s[29].

### Study population

Participants in this study were all traditional healers and members of the Kukula Organization, an educational and advocacy group based in the Agincourt area. We used a random number generator to recruit participants from the top quartile of active healers from a previous study – which included a random sample 221 of 300 healers in Agincourt – on HIV counseling and testing [17]. All successfully contacted healers (30 of 54; 55% via phone and in-person visits) were interviewed (100% acceptance rate) and all healers were over the age of 18. Our goal was to continue interviewing participants until we reached data saturation [30]. We reached saturation at interview 26, but due to the lag time in transcription did not realize this until we had 30 interviews transcribed.

### Ethics, consent and permissions

This study was approved by Vanderbilt’s Institutional Review Board (IRB #190395), University of Witwatersrand’s Human Research Ethics Committee (Medical) (Protocol #M160447), as well as Mpumalanga Department of Health’s Research Ethics Committee. All participants provided written informed consent in their preferred language.

### Data collection

Between April and June 2019, we (SN, and MM) conducted 30 semi-structured in-depth, in-person interviews at the homes of the traditional healer. The interview guide used a mix of open and closed ended questions which were asked of all healers in the same order in each interview see supplemental file 1). Quantitative data such as number of patients treated in past 6 months, years in practice and education level were collected at the beginning of the interview. Qualitative questions focused on healer attitudes and practices in regards to glove use during traditional treatments – including patient baths, injections, or other treatments that exposed healers to patient blood or open sores.

Questions were developed based on responses to surveys completed the previous year. This guide was piloted with three healers and two fieldworkers in Agincourt. Two trained qualitative fieldworkers (female) conducted one-on-one interviews in xiTsonga and took place at the home or other preferred meeting place of the traditional healer. These interviewers have undergraduate degrees in education and public health, respectively, and have received training in qualitative methods while working with several NIH-funded research projects over the past 8 years. The participants had previously met the interviewers during the parent survey where they were tested for HIV and during community feedback sessions (in 2018). Participants knew we were interested in their risk to HIV during exposure to patient blood during traditional treatments, given our focus on HIV-testing among this population. Field notes were taken during the interview. After being audio-recorded, interviews were transcribed and translated to English from xiTsonga. Interviews averaged 27 min in duration.

### Data analysis

xiTsonga interviews were transcribed immediately following their recording, and translated into English by a qualified fieldworker who was not involved in the interviewing process. Translation was verified by a second fieldworker fluent in both languagesEnglish transcripts were reviewed both by the interviewers and by researcher EMC upon their completion, and two researchers (EMC and CMA) conducted thematic analysis using MAXQDA 12© software. This analysis occurred on a rolling basis as the interview transcripts were completed. Previous research about healer engagement in the health system and challenges implementing latex glove use among health care workers [31] along with the information-motivation-behavior theory [32–34] guided deductive code development, while in vivo (inductive) codes were generated when unique benefits or challenges to obtaining or using gloves were identified. EMC and CMA met to develop, define, and compare application of codes to the transcribed interviews; after which, there was complete agreement of deductive and inductive codes and subcodes to the 30 interviews. The results of the study were discussed with the healers as a group, but not with individual healers in the study, for feedback.

## Results

The traditional healers interviewed had a median age of 52 (Interquartile Range [IQR] 45–59), low levels of formal education (median: 5 years, IQR: 0–8), and were 70% female. The 30 healers interviewed practiced in 13 villages and reported a median of 17 years in practice (IQR 7.3–22.8).

### Traditional healers desire to protect themselves from blood exposure

All interviewed healers expressed an understanding that HIV can be transmitted through contact with the bodily fluids of infected persons. Further, all except one (97%) expressed concern that they may be exposed to patient blood, and therefore diseases, during their treatment encounters,

*“It worries me because if I vaccinate the patients without using the gloves I may get infected with diseases through the process of vaccination. I may have infectious diseases or the patient has them and we may infect each other in the process if I don’t use the hand gloves.” (male, 30–39).*

The single traditional healer who did not fear exposure did describe some glove use, however she lacked concern about becoming infected, stating, “when I do the vaccination, I just don’t care whether I get infected or not because I tell myself that it is my job” (female, 40–49).

Overwhelmingly, participants believed gloves were safe, reliable and protected them from diseases – including HIV-, attributing this knowledge to lessons from researchers from the University of the Witwatersrand (who work with the HDSS in the area) and clinicians who work at local health facilities.

### Healers face challenges to consistent, adequate PPE usage

While 90% of healers reported using latex gloves during some treatments, their understanding of the benefits did not translate into regular use (Fig. 1). Free gloves were previously distributed to many of the healers by a nurse at one local health facility given her personal interest in improving the relationship between healers and the health system, but that nurse recently passed away. One woman lamented this, “I used to get gloves from a certain nurse who is yet no more and I was using those gloves to protect myself” (female, 60–69).

Healers now must purchase their own gloves at local pharmacies. When given the gloves, healers demonstrated the desire to use them. However, healers cited the high cost of the gloves (50%), distance to the pharmacy (37%), and pharmacies being out of stock (6.7%) as barriers to appropriate glove use. To overcome these challenges, most participants suggested that the government provide gloves. Few were willing to use their own money to access them (local cost per glove is approximately 0.16 USD); in part due to poverty (healers make an average of 272 USD per month) and in part due to the belief that the system should provide gloves for their safety.

*The government should give the healers the hand gloves because we are struggling and we are at risk of contracting the diseases when we don’t have the hand gloves. Traditional healing is another way of treating patients and we have some things in common with the medical doctors because we also inspect the wounds and treat them, and that requires the gloves. (female, 70–79).*

Many healers saw their work as a partnership with the health system, given that they refer patients to the allopathic health system for various tests and treatments.

*I would be really happy* [if we were given gloves from the clinic] *because we work hand in hand with the clinics and if I have patients that the medical attention, I am able to refer them to the clinic. For instance, if a patient comes with dehydration, I have to refer that patient to the clinic. (male, 40–49).*

Only3 healers reported always using latex gloves during blood exposure or open wounds in the past month. Only one respondent used latex gloves donated from a local health facility. The other two healers chose to purchase their own supply of gloves when necessary. One of these ‘early adopter’ healers explained her decision:

*… the safe way to prevent the diseases from being contracted from the patient is to wear the gloves at all times when I am doing the vaccinations. The gloves keep the both of us safe from diseases. But I don’t like it when I have run out of the gloves and I make sure that when they are about to be finished, I go and buy more.* (female, 50–59).

These early adopters may be the key to convincing their colleagues to use protection regularly.

### Current protective practices are resourceful yet inadequate

Healers were concerned with blood exposure: 93% believed gloves would protect them from contracting HIV from their patients, but glove availability remains a challenge. Most employed a combination of gloves, plastic shopping bags, bread bags, paper, and sticks to prevent blood exposure. One healer said,

*When a patient comes for vaccinations, I prepare the medicine first, wear the gloves, then open a razor blade and start with the vaccinations … When I don’t have the hand gloves, I use the plastics [shopping bags]. I never vaccinate without wearing anything on my hands*. (female, 50–59).

Healers reported plastic bags slipping or breaking during procedures, exposing them to patient blood.

*I put my hands in the plastics and tie them around my hands and use my fingers to apply the medicines, but the plastics sometimes slips through my hands and I accidentally touch the patients’ blood.* (male, 60–69).

Three traditional healers reported never using gloves during treatments. These healers try to avoid blood exposure via use of bread bags, shopping bags and smooth sticks to apply the herbs. One woman explained, “I use a small stick to apply the medicines on the cuts to ensure that I don’t touch the blood with my bare hands, other than that; there is nothing else that I use for protection.” (Female, 60–69).

## Discussion

Inadequate PPE use and high HIV prevalence make traditional healers particularly susceptible to contracting HIV in rural South Africa [17]. Among participating healers, a majority expressed a willingness to use gloves, believed they were at high risk of infection through blood exchange, and indicated fairly good relations with the local department of health – a potential source of gloves. Despite positive attitudes, consistent glove use remains low due to financial constraints, glove availability, and distance to clinics and pharmacies (where they can be acquired). Only three healers (10%) were willing to pay for their own gloves to ensure that they were protected. These three “early adopter” healers were motivated by two things: (1) a clear understanding of the importance of gloves to prevent the spread of disease and (2) a belief that they trusted, and were in partnership, with the allopathic health system.

Addressing issues of accessibility and cost of gloves for traditional healers could have a significant impact on the adherence to PPE and, in turn, reduce new HIV infections among this high-risk group. A concerted effort by the South African Department of Health to provide healers with gloves, subsidize glove costs, or incentivize glove use is one solution, but the the extensive nextwork of traditional healers around the country (~ 200,000) makes this an expensive proposition.

Another potential option would be for healers to require patients to bring a new set of gloves (along with the razor blade) for the procedure. Healers already employ this successful strategy with the razor blades used in procedures after a long campaign by the government to encourage new blade use [35]. Patients were sensitized about the importance of using a new blade for each procedure to prevent disease transmission and healers placed the onus for blade procurement on the patient. Although there was initially minor patient resistance, razor blades are easily accessible and inexpensive. One barrier to employing this strategy is that gloves are typically sold by the box; pharmacies and health facilities would have to adjust to selling gloves by the pair.

The availability of gloves and accurate identification of risk does not eliminate the concern that healers may be incorrectly using PPE. Healers need to develop skills to effectively don, doff, and dispose of used PPE. We suggest training led by health care workers and/or by “early-adopter” healers who are using PPE regularly and correctly. We hypothesize that “early adopter” healer messaging and delivery will have greater impact on healer behavior given their trusted position within their own community, will be more easily scaled up nationwide given the large number of healers in SA, and will be conducted more cost-efficiently given the lower hourly rate for healers (vs. health care workers).

Lastly, strategies for the disposal of used PPE is an important concern, given that healers are re-using gloves/plastics or leaving them around the yard after treatment is complete. Disposal strategies need to be tailored to the healer location: those in rural locations can safely put them in the latrine while those in urban areas need a safe place to dispose of them. If provided a container, healers could store used razor blades and gloves until they visit a health facility for disposal.

Our study results are limited by the small sample size. Our small number of interviewees (30) allowed for greater depth of information, including individual, social, and institutional factors that motivate and/or limit PPE use. It is possible that we did not reach a certain sub-set of healers (perhaps those who are not concerned about disease transmission) given that we recruited via the local healer association. Additionally, we recruited healers who saw the largest number of patients; those who see fewer patients may have different views on HIV transmission risk or use PPE in different ways than those who are regularly being exposed to patient blood. Lastly, in the parent study, we made efforts to reach those healers who have varying levels of contact with the health system, but those with no contract at all would not be have previously identified during survey and/or outreach activities. Thus, there may be a group of healers unwilling to work with the allopathic system that we did not access during our interviews.

## Conclusion

Traditional healers are generally aware of the risks associated with blood exposure and undertake various strategies to protect themselves. Despite this knowledge, PPE use is inconstant, and healers desire to protect their own health does not overcome the financial considerations associated with buying latex gloves. The development of training and messaging strategies, along with the increasing the availability of low-cost or free gloves, could protect this large, high risk population in rural South Africa.

## Data Availability

The datasets generated and/or analyzed during the current study are not publicly available because they contain information that could compromise research participant privacy/consent but are available from the corresponding author on reasonable request.

## Abbreviations

HDSS: Health and Socio-Demographic Surveillance Survey
HBV: Hepatitis B Virus
HCV: Hepatitis C Virus
HIV: Human Immunodeficiency Virus
NIH: National Institutes of Health
PPE: Personal protective equipment
SSA: Sub-Saharan Africa

## References

[1] South Africa [https://www.unaids.org/en/regionscountries/countries/southafrica].

[2] Key Populations [https://www.unaids.org/en/topic/key-populations].

[3] Karani H, Rangiah S, Ross AJ (2011). Occupational exposure to blood-borne or body fluid pathogens among medical interns at Addington hospital. Durban South African Family Practice.

[4] Rossouw TM, van Rooyen M, Louw JM, Richter KL: Blood-borne infections in healthcare workers in South Africa. South African medical journal = Suid-Afrikaanse tydskrif vir geneeskunde 2014, 104(11):732–735. 10.7196/samj.8518 25909108

[5] Patel P, Borkowf CB, Brooks JT, Lasry A, Lansky A, Mermin J (2014). Estimating per-act HIV transmission risk: a systematic review. AIDS.

[6] Audet CM, Blevins M, Moon TD (2012). M S, shepherd BE, Pires P, Vergara AE, Vermund SH: HIV/AIDS-related attitudes and practices among traditional healers in Zambezia Province, Mozambique. Journal of Alternative and Complementary Medince.

[7] Peters EJ, Immananagha KK, Essien OE, Ekott JU (2004). Traditional healers' practices and the spread of HIV/AIDS in south eastern Nigeria. Trop Dr.

[8] Wojcicki JM, Kankasa C, Mitchell C, Wood C (2007). Traditional practices and exposure to bodily fluids in Lusaka, Zambia. Tropical medicine & international health : TM & IH.

[9] Audet CM, Salato J, Blevins M, Silva W, Gonzalez-Calvo L, Vermund SH, Gaspar F (2016). Occupational hazards of traditional healers: repeated unprotected blood exposures risk infectious disease transmission. Tropical medicine & international health : TM & IH.

[10] Traditional Medicines and Traditional Healers in South Africa. [http://www.tac.org.za/Documents/ResearchPapers/Traditional_Medicine_briefing.pdf].

[11] Government Gazette: Traditional Health Practitioners Act, 2007. In., vol. 511. Cape Town; 2008.

[12] Jolles F, Jolles S (2011). Zulu ritual immunisation in perspective. Africa.

[13] Scorgie F, Beksinska M, Chersich M, Kunene B, Hilber AM (2010). Smit J: “cutting for love”: genital incisions to enhance sexual desirability and commitment in KwaZulu-Natal. South Africa Reproductive Health Matters.

[14] Audet CM, Ngobeni S, Wagner RG (2016). Use of CD4+ cell count results to determine traditional treatment eligibility among HIV-infected patients in rural South Africa. In: AIDS 2016. Vol. WEPDD0102.

[15] Audet CM, Blevins M, Rosenberg C, Farnsworth S, Salato J, Fernandez J, Vermund SH: Symptomatic HIV-positive persons in rural Mozambique who first consult a traditional healer have delays in HIV testing: a cross-sectional study. Journal of acquired immune deficiency syndromes (1999) 2014, 66(4):e80–86. 10.1097/QAI.0000000000000194 PMC4077983 24815853

[16] Gomez-Olive FX, Angotti N, Houle B, Klipstein-Grobusch K, Kabudula C, Menken J, Williams J, Tollman S, Clark SJ (2013). Prevalence of HIV among those 15 and older in rural South Africa. AIDS Care.

[17] Audet CM, Ngobeni S, Mkansi M, Wafawanaka F, Aliyu MH, Kahn K, Wagner R: Occupational hazards of traditional healers in rural South Africa: Bloodborne pathogen exposures and risk of HIV transmission. In: 22nd International AIDS Conference Amsterdam, Netherlands; 2018.

[18] Howard DP, Williams C, Sen S, Shah A, Daurka J, Bird R, Loh A, Howard A (2009). A simple effective clean practice protocol significantly improves hand decontamination and infection control measures in the acute surgical setting. Infection.

[19] Phin NF, Rylands AJ, Allan J, Edwards C, Enstone JE, Nguyen-Van-Tam JS (2009). Personal protective equipment in an influenza pandemic: a UK simulation exercise. The Journal of hospital infection.

[20] Bingham J, Abell G, Kienast L, Lerner L, Matuschek B, Mullins W, Parker A, Reynolds N, Salisbury D, Seidel J (2016). Health care worker hand contamination at critical moments in outpatient care settings. Am J Infect Control.

[21] Jain S, Clezy K, McLaws ML (2019). Modified glove use for contact precautions: health care workers' perceptions and acceptance. Am J Infect Control.

[22] Loveday HP, Lynam S, Singleton J, Wilson J (2014). Clinical glove use: healthcare workers' actions and perceptions. The Journal of hospital infection.

[23] Kaczmarek RG, Moore RM, McCrohan J, Arrowsmith-Lowe JT, Caquelin C, Reynolds C, Israel E (1991). Glove use by health care workers: results of a tristate investigation. Am J Infect Control.

[24] Rudolph MJ, Ogunbodede EO: HIV infection and oral health care in South Africa. SADJ : journal of the South African Dental Association = tydskrif van die Suid-Afrikaanse Tandheelkundige Vereniging 1999, 54(12):594–601. 16892566

[25] Kahn K, Collinson MA, Gomez-Olive FX, Mokoena O, Twine R, Mee P, Afolabi SA, Clark BD, Kabudula CW, Khosa A (2012). Profile: Agincourt health and socio-demographic surveillance system. Int J Epidemiol.

[26] Tollman SM: The Agincourt field site--evolution and current status. South African medical journal = Suid-Afrikaanse tydskrif vir geneeskunde 1999, 89(8):853–858. 10488361

[27] Tollman SM, Herbst K, Garenne M, Gear JS, Kahn K: The Agincourt demographic and health study--site description, baseline findings and implications. South African medical journal = Suid-Afrikaanse tydskrif vir geneeskunde 1999, 89(8):858–864. 10488362

[28] Tollman SM, Kahn K, Garenne M, Gear JS (1992). Reversal in mortality trends: evidence from the Agincourt field site, South Africa. AIDS (London, England) 1999.

[29] Audet CM, Ngobeni S, Wagner RG (2017). Traditional healer treatment of HIV persists in the era of ART: a mixed methods study from rural South Africa. BMC Complement Altern Med.

[30] Guest G, Bunce A, Johnson L (2006). How many interviews are enough? An experiment with data saturation and variability. Field Methods.

[31] Honda H, Iwata K (2016). Personal protective equipment and improving compliance among healthcare workers in high-risk settings. Curr Opin Infect Dis.

[32] Amico KR (2011). A situated-information motivation behavioral skills model of care initiation and maintenance (sIMB-CIM): an IMB model based approach to understanding and intervening in engagement in care for chronic medical conditions. J Health Psychol.

[33] Fisher CM (2011). Are information, motivation, and behavioral skills linked with HIV-related sexual risk among young men who have sex with men?. J HIV AIDS Soc Serv.

[34] Fisher JD, Amico KR, Fisher WA, Harman JJ (2008). The information-motivation-behavioral skills model of antiretroviral adherence and its applications. Current HIV/AIDS reports.

[35] Ayisi R (2006). Traditional healers' practices under AIDS spotlight.

